# Heavy Metal Stress Alters the Response of the Unicellular Cyanobacterium *Synechococcus elongatus* PCC 7942 to Nitrogen Starvation

**DOI:** 10.3390/life10110275

**Published:** 2020-11-07

**Authors:** Khaled A. Selim, Michael Haffner

**Affiliations:** Organismic Interactions Department, Interfaculty Institute for Microbiology and Infection Medicine, Cluster of Excellence ‘Controlling Microbes to Fight Infections’, Tübingen University, Auf der Morgenstelle 28, 72076 Tübingen, Germany; michael.haffner@student.uni-tuebingen.de

**Keywords:** heavy metal stress, chlorosis, resuscitation, non-diazotrophic cyanobacteria, *Synechococcus elongatus* PCC 7942

## Abstract

Non-diazotrophic cyanobacteria are unable to fix atmospheric nitrogen and rely on combined nitrogen for growth and development. In the absence of combined nitrogen sources, most non-diazotrophic cyanobacteria, e.g., *Synechocystis* sp. PCC 6803 or *Synechococcus elongatus* PCC 7942, enter a dormant stage called chlorosis. The chlorosis process involves switching off photosynthetic activities and downregulating protein biosynthesis. Addition of a combined nitrogen source induces the regeneration of chlorotic cells in a process called resuscitation. As heavy metals are ubiquitous in the cyanobacterial biosphere, their influence on the vegetative growth of cyanobacterial cells has been extensively studied. However, the effect of heavy metal stress on chlorotic cyanobacterial cells remains elusive. To simulate the natural conditions, we investigated the effects of long-term exposure of *S. elongatus* PCC 7942 cells to both heavy metal stress and nitrogen starvation. We were able to show that elevated heavy metal concentrations, especially for Ni^2+^, Cd^2+^, Cu^2+^ and Zn^2+^, are highly toxic to nitrogen starved cells. In particular, cells exposed to elevated concentrations of Cd^2+^ or Ni^2+^ were not able to properly enter chlorosis as they failed to degrade phycobiliproteins and chlorophyll a and remained greenish. In resuscitation assays, these cells were unable to recover from the simultaneous nitrogen starvation and Cd^2+^ or Ni^2+^ stress. The elevated toxicity of Cd^2+^ or Ni^2+^ presumably occurs due to their interference with the onset of chlorosis in nitrogen-starved cells, eventually leading to cell death.

## 1. Introduction

Cyanobacteria occupy a privileged position in the Earth’s history as a key player in global C/N cycles [1] and as inventors of oxygenic photosynthesis by evolving two coupled photosystems. Thereby, they enriched the Earth’s atmosphere with oxygen around 2.4 billion years ago [2]. Physiologically, with respect to nitrogen demand, cyanobacteria can be classified into two distinct groups: (1) diazotrophic cyanobacteria, which can fix atmospheric gaseous nitrogen via an enzyme called nitrogenase (e.g., *Anabaena variabilis* PCC 7937 and *Nostoc* sp. PCC 7120 of heterocyst-forming cyanobacteria and *Cyanothece* sp. 51142 of unicellular non-heterocystous cyanobacteria) [3,4], and (2) non-diazotrophic cyanobacteria, which are unable to fix atmospheric nitrogen and rely on the availability of a combined nitrogen source, such as nitrate or ammonia, for growth and intracellular catabolic and anabolic reactions (e.g., *Synechocystis* sp. PCC 6803 and *Synechococcus elongatus* PCC 7942) [1,5].

In nature, nitrogen availability is highly variable. Under environmental depletion of combined nitrogen, most non-diazotrophic cyanobacteria respond by degrading their photosynthetic pigments, in a process termed chlorosis [6]. Upon prolonged starvation, they switch to a maintenance lifestyle by tuning down their anabolic processes. Later on, they enter a dormant state, where the chlorotic cells survive the prolonged periods of nitrogen starvation [7]. The chlorosis process involves degradation of phycobilisomes (light-harvesting complex), chlorophyll a, the bulk of cellular proteins, and thylakoid membranes; downregulation of protein biosynthesis and energy-consuming reactions; accumulation of reserve polymers of glycogen and poly-β-hydroxybutyrate (PHB) [5,8]. A hallmark of chlorosis is the cell cycle arrest and the turn from a blue-green color to a yellow color due to the degradation of the photosynthetic apparatus machinery [8,9]. A minimal residual photosynthesis [8,10] (~0.1%) is, however, maintained to retain cell viability over prolonged periods up to 6 months.

Upon re-availability of combined nitrogen, the chlorotic dominant cyanobacteria rapidly awake and return greenish to the vegetative growth in a process called resuscitation [9]. Therefore, resuscitation can be considered as the reverse process of chlorosis and takes place through two phases [8,9]. Within the first 12–16 h (the first phase of resuscitation), cells reactivate their protein biosynthesis and machinery for nitrate assimilation (*narB*; *nir* operon; *moa* gene cluster for the molybdenum co-factor of nitrate reductase) and rebuild the entire F-type ATPase machinery [5]. The required energy within the first phase of resuscitation is generated by glycogen degradation [11]. In the second phase of resuscitation, a metabolic rewiring towards the photosynthetic machinery takes place, including re-pigmentation, re-building of thylakoid membranes and re-generation of photosystem II and oxygen evolution activities [8,9]. The reconstitution and recovery of the photosynthetic apparatus is recognizable after 24 h of resuscitation, as evidenced by re-greening of the cells and increased photosystem II activity, while proper cell growth is first detectable after 48 h [8].

Cyanobacteria have specific needs for metals, which frequently differ from those of other bacteria—Mn^2+^ is required for chlorophyll biosynthesis and for water-splitting oxygen-evolving complex, Cu^2+^ for plastocyanin and cytochrome oxidase, Zn^2+^ for DNA and RNA polymerases as well as for the carboxysome-localized carbonic anhydrase (for carbon assimilation), Co^2+^ for vitamin B_12_ biosynthesis and Mo^2+^ for nitrogenase in heterocysts [12,13,14]. Those metals are considered as essential elements and required in trace amounts, while non-essential metals (e.g., Cd^2+^, Pb^2+^, Cr^2+^ and Hg^2+^) are toxic to cyanobacteria [13,14]. In addition, the essential metals are more generally required to provide defense against oxidative stresses, transfer electrons in photosynthetic reaction centers and serve as cofactors for metalloproteins. However, high concentrations of heavy metals, irrespective of whether they are essential or not, negatively influence cyanobacterial metabolism. Mostly affected is the photosynthetic machinery with alterations in the photosynthetic electron transport chain, several photosynthetic enzymes, dark reactions, ATP synthesis and photosynthetic pigments, leading to photoinhibition. For example, Pb^2+^, Mn^2+^ and Cd^2+^ can inhibit chlorophyll a and b biosynthesis [13,14].

Based on mutational analysis in *Escherichia coli*, the CutA protein (**Cu**^2+^ **t**olerance protein **A**) has been proposed to be implicated in bacterial divalent ion tolerance. However, we recently were unable to link CutA to heavy metal tolerance in cyanobacteria under vegetative growth [15]. As heavy metals are ubiquitous in the cyanobacterial biosphere, a proper control of metal homeostasis is, therefore, essential for the photosynthetic lifestyle of cyanobacteria. While nitrogen remains the growth-limiting factor, other environmental factors, such as heavy metals, could be toxic to the vegetative cells owing to their interference with vital intracellular processes [13,14]. However, it is unclear how they affect the cyanobacteria when they are in the dormant chlorotic state. Since nitrogen limitation is a frequent environmental stress, we aimed to reveal how heavy metal stress and nitrogen limitation could affect wild-type and ∆*cutA* cells in the cyanobacterium model organism, *S. elongatus* PCC 7942.

## 2. Materials and Methods 

### 2.1. Microbiology Biology Methods 

Exponentially growing wild-types of either *Synechococcus elongatus* PCC 7942 and Δ*cutA* mutant cells of OD_750_ 0.3–0.5 were subjected to heavy metals stress by supplementing unbuffered BG11 media with one of the following heavy metals: PbCl_2_, CrCl_2_, MnCl_2_, ZnCl_2_, CuSO_4_, NiCl_2_ and CdCl_2_ in concentrations from 2.5 to 50 µM in a 24-well plate. For chlorosis experiments, the heavy metal treatments were added to BG11_0_ (BG11 media without nitrate) to induce metal stress and chlorosis [8] for *S. elongatus* PCC 7942 and *ΔcutA* mutant of an initial OD_750_ 0.5. The survival of nitrogen-starved/heavy-metal-stressed cells was checked by drop assay [15,16], where 5 µl cells from each treatment was spotted on nitrate-supplemented BG11 agar plates in the absence of heavy metals and incubated at 28 °C under a light intensity of 30–50 µmol photons m^−2^ s^−1^ for one week.

### 2.2. Pulse-Amplitude-Modulation (PAM) Measurements

The photosynthetic fitness for the recovery of the nitrogen-starved/heavy-metal-treated cells was estimated by measuring photosystem II (PSII) activity under a 50 µE light intensity using WATER-PAM chlorophyll fluorescence (Walz GmbH), as described previously [16,17]. The maximal PSII quantum yield was determined with the saturation pulse method using the *F0−Fm/Fm* ratio [17].

### 2.3. Photosynthetic Oxygen Evolution Measurements

In vivo photosynthetic oxygen evolution was estimated using an oxygen electrode of the Clark-type (Hansatech DW1) [16,17]. Oxygen evolution of 2 mL of recovering cultures normalized to an OD_750_ of 0.4 was measured at room temperature at 50 μE. The 50 µE light intensity was provided using a high-intensity white-light source (Hansatech L2). 

### 2.4. Molecular Biology Methods

Transformation of *S. elongatus* PCC 7942 to create a knockout mutant in the open reading frame (ORF *Synpcc7942_2261*) encoding for CutA was achieved by replacement of the ORF *Synpcc7942_2261* by kanamycin resistance cassette, as described previously for *Synechocystis* sp. PCC 6803 [16]. The *ΔcutA* mutant was selected on BG11 plates supplemented with 50 µg/ml kanamycin and verified with PCR, as shown previously elsewhere [15].

## 3. Results

To simulate the natural situation and to study the influence of long-time exposure of chlorotic cells to heavy metals, we combined heavy metal stress and nitrogen starvation on *S. elongatus* wild-type (WT) and *cutA* null mutant (∆*Se*CutA) cells, which we previously showed not to be involved in heavy metal tolerance in vegetative growing cyanobacteria [15]. The ∆*Se*CutA mutant was generated by replacement of the *cutA* encoding gene *synpcc7942_2261* with a kanamycin-resistant gene in *S. elongatus* PCC 7942 [15].

**Figure 1:**
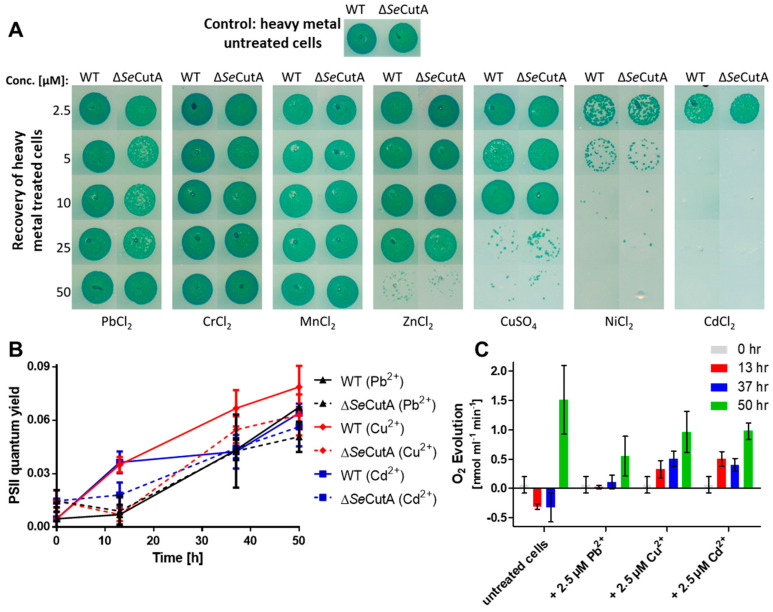
Influence of heavy metal treatments on the chlorotic *S. elongatus* WT and ∆*Se*CutA cells. (**A**) The survival of nitrogen-starved/heavy-metal-treated cells (as indicated) was evaluated by drop assay on BG11 (nitrate-supplemented, in absence of heavy metals) media. (**B**,*C*) Shown are the PSII quantum yield (**B**) and photosynthetic oxygen evolution (**C**) for the recovery of *S. elongatus* cells over 50 h of resuscitation from a long period (28 days) of chlorosis and 2.5 µM heavy metal treatment (as indicated). Both PSII quantum yield in (**B**) and oxygen evolution in (**C**) were determined under a light intensity of 50 µE, using pulse-amplitude-modulation (PAM) fluorometry and an oxygen electrode, respectively.

When exposed to low concentrations of heavy metals (2.5 µM) and nitrogen starvation, the WT and ∆*cutA* cells were able to resuscitate on nitrate-supplemented BG11 plates lacking heavy metals (Figure 1A; first row). These results were confirmed further by measuring the recovery of the photosynthetic apparatus (photosystem II; PSII) using pulse-amplitude-modulation (PAM) fluorometry for WT and ∆*cutA* cells, resuscitating from a long period (28 days) of chlorosis in the presence of 2.5 µM heavy metals (PbCl_2_, CuSO_4_ or CdCl_2_). Over 50 h of resuscitation, the efficiency of PSII regeneration for WT and ∆*cutA* cells was comparable (Figure 1B). Additionally, in agreement with the increase in PSII quantum yield in Figure 1B, photosynthetic oxygen evolution of the same cells was also recognizable after 50 h of resuscitation (Figure 1C). However, the net oxygen evolution of heavy-metal-treated cells was reduced by 30 to 40% in comparison to untreated cells, implying that the cells were severely stressed by the presence of all the heavy metals tested but were still able to recover. A similar phenotype was observed for the ∆*cutA* mutant. This observation motivated us to check further for the influence of higher concentrations of heavy metals on chlorotic cells.

Interestingly, when *S. elongatus* cells were nitrogen-starved for 5 days and simultaneously exposed to different heavy metals (Pb^2+^, Cr^3+^, Mn^2+^, Zn^2+^, Cu^2+^, Ni^2+^ and Cd^2+^) at concentrations higher than 2.5 µM, different toxicities were observed (Figure 1A). Recovery of treated cells on BG11 plates (nitrate-supplemented, in absence of heavy metals) revealed a high toxicity of Ni^2+^ and Cd^2+^ ions to chlorotic cells, already apparent at the concentration of 5 µM, whereas Cu^2+^ and Zn^2+^ ions showed intermediate toxicity and the rest of the tested ions showed no toxicity (Figure 1A). A similar pattern of toxicity was observed for the ∆*cutA* mutant (Figure 1A).

**Figure 2:**
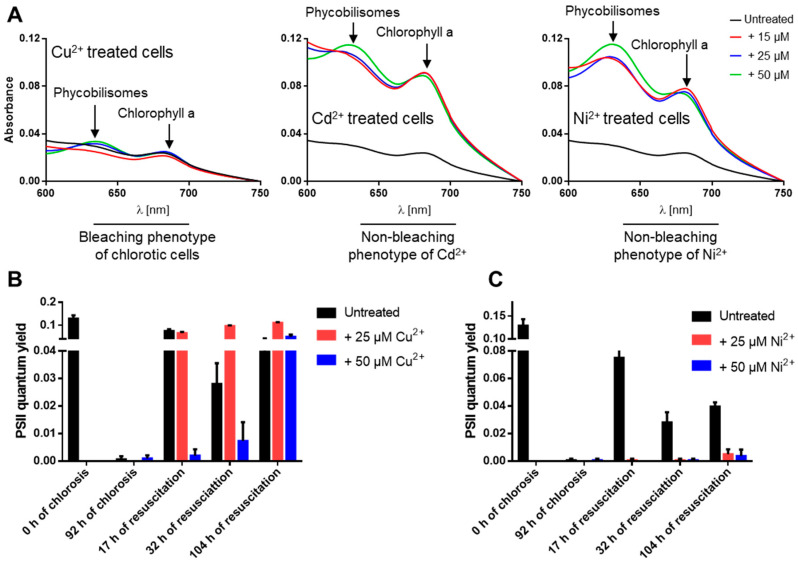
The non-bleaching phenotype of nitrogen-starved and heavy-metal-treated *S. elongatus* cells leads to cell death. (**A**) Absorption spectra of nitrogen-starved and heavy-metal-treated cells as indicated, revealing impair degradation of phycobiliproteins and chlorophyll a under Cd^2+^ and Ni^2+^ treatment. (**B***,***C**) The PSII quantum yield for the recovery of resuscitated cells from chlorosis and high metal concentrations (25 or 50 µM) of Cu^2+^ (**B**) or Ni^2+^ (**C**) by PAM fluorometry under a light intensity of 50 µE.

During normal nitrogen starvation-induced chlorosis, the cells turn from a deep blue-green to a yellowish color due to NblA-induced pigment degradation [5,8]. Surprisingly, we observed that under nitrogen starvation conditions and in the presence of elevated levels of the heavy metals Ni^2+^, Cd^2+^, Cu^2+^ and Zn^2+^, the cells remained green due to impaired degradation of phycobiliproteins and chlorophyll a (visible in absorbance changes at 625 and 680 nm) and were not able to enter chlorosis properly (Figure 2A). These non-bleaching cells died after several days of heavy metal stress and nitrogen starvation as indicated by the inability of the cells to recover on BG11 (Figure 1A). To confirm these results, PAM fluorometry was used to monitor (over 104 h) the PSII activity of cells recovering from a 92-hour-long treatment with high concentrations (25 and 50 µM) of either medium toxic (Cu^2+^) or high toxic (Ni^2+^) ions during nitrogen starvation (Figure 2B,C). This analysis revealed that PSII activity resumed in cells that were recovering from Cu^2+^ treatment during chlorosis (Figure 2B), confirming the low toxicity of Cu^2+^ ions to the chlorotic cells. By contrast, cells that had been exposed to elevated concentrations of Ni^2+^ during chlorosis were not able to recover after the shift to nitrogen-rich and Ni^2+^-free media (Figure 2C), confirming the high toxicity of Ni^2+^ ions and the death of the chlorotic cells after NiCl_2_ treatment.

## 4. Discussion

Nitrogen-starved WT and *ΔcutA* cells were exposed to different concentrations of heavy metals to check the toxicity of heavy metals on chlorotic cells (Figure 1A,B). Our results, presented here, clearly reveal that elevated heavy metal concentrations, especially for Ni^2+^, Cd^2+^, Cu^2+^ and Zn^2+^, are severely toxic to nitrogen-starved cells. Our data suggest that this is most probably due to dysregulation of the bacterium metabolism and the highly oxidative stress imposed on the cells. In particular, cells exposed to elevated concentrations of Cd^2+^ or Ni^2+^ failed to enter chlorosis properly as they were not able to degrade phycobilisomes and chlorophyll a (Figure 2A). In resuscitation assays, these cells were able to recover from chlorosis coupled with Cu^2+^ or Zn^2+^ stress but not Cd^2+^ or Ni^2+^ stress (Figure 1A and Figure 2B,C). Therefore, we concluded that the elevated toxicity of Cd^2+^ or Ni^2+^ occurs due to their interference with the onset of chlorosis in nitrogen-starved cells, which finally leads to cell death, most likely due to accumulation of reactive oxygen species.

The *ΔcutA* mutant was shown, previously, not to be involved in heavy metal stress in cyanobacteria [15]. In agreement with our previous results [15], we were unable to link the *S. elongatus ΔcutA* mutant to heavy metal tolerance even under severe stress conditions by combining heavy metal and nitrogen stress (Figure 1A,B). Indeed, we found no difference between the *S. elongatus cutA* mutant and wild-type strains recovering from heavy metal stress either in the presence [15] or absence of combined nitrogen sources, confirming that CutA plays a different role in cyanobacteria rather than participating in heavy metal resistance.

As photoautotroph microbes, cyanobacteria are considered promising microbial factories for sunlight- and CO_2_-fueled production of a vast array of high-value chemicals (e.g., biopolymers) that are of remarkable interest for industry and human health. Synthetic biology strategies to engineer cyanobacteria rely on robust promoters with predictable input−output responses to tightly control the desired gene expression for sustainable biotechnological applications [18]. Among the promoters used to engineer cyanobacteria, several are metal-inducible promoters (e.g., Cu^2+^-dependent P*_petJ_* and P*_petE_*, Ni^2+^-dependent P*_nrsB_*, Zn^2+^-dependent P*_zia_* and P*_smt_*, Co^2+^-dependent P*_coaT_*, and P*_copM_* which is induced by Ni^2+^, Cd^2+^, Cu^2+^ and Zn^2+^) [19]. The Ni^2+^-inducible P*_nrsB_* promotor was shown to be among the most strong and versatile promoters in the unicellular cyanobacterium *Synechocystis* sp. PCC 6803, with expression levels nearly up to the activity of the strongest *Synechocystis* promotor, P*_psbA2_* [20,21]. However, the bottleneck in using metal-dependent promoters in cyanobacterial synthetic biology applications is the metal toxicity at higher concentrations, especially for Cu^2+^, Ni^2+^, Zn^2+^ and Co^2+^ [12,18,19,20].

Some of useful cyanobacterial products accumulate under nitrogen starvation conditions—for example, PHB [22]. As a biopolymer, PHB receives, nowadays, a lot of attention for biotechnological applications as a replacement of conventional plastic. Consistent with our results, attempts to overexpress the PHB synthase-encoding gene in *Synechocystis* sp. PCC 6803 under the control of the strong Ni^2+^-inducible promotor in nitrogen-depleted conditions were unsuccessful [23]. Our data suggest that this is likely due to the high toxicity of Ni^2+^ under nitrogen starvation. Furthermore, recent studies revealed a limitation in the use of the P*_nrsB_*, P*_petJ_* and P*_coaT_* promoters for biotechnological applications in cyanobacteria at high metal concentrations [18,20,24]. All of these observations hinder the use of metal-dependent promoters for synthetic biology applications in cyanobacteria, especially under nitrogen starvation conditions.

Collectively, this study sheds light on an unexplored area of cyanobacteria physiology and serves as a valuable guide for synthetic biology approaches in cyanobacteria by defining the clear limitation of using heavy metal-inducible promoters under nitrogen starvation. A recent proteomic study defined the global proteomic response of *Synechocystis* sp. PCC 6803 to Cu^2+^ applied to regulate the Cu^2+^-inducible promoter P*_petJ_* [24]. Notably, they identified clear irreversible proteomic changes due to Cu^2+^ stress, including significant alternations in the protein amounts of the outer and inner membranes and of the cell surface and the downregulation of ribosomal proteins.
